# Maternal Tributyrin Supplementation During the Perinatal Period Is Associated with Improved Ewe Milk Quality and Lamb Growth Performance, Immunity, and Antioxidant Status

**DOI:** 10.3390/vetsci13030231

**Published:** 2026-02-28

**Authors:** Xu-Nan Gao, Xin-Le Zhang, Jian-Xin Zuo, Yuan-Xiao Wang, Pei-Yong Chen, Yan-Li Zhang, Feng Wang, Yi-Xuan Fan

**Affiliations:** 1 Institute of Sheep and Goat Science, Nanjing Agricultural University, Nanjing 210095, China; gaoxunan@stu.njau.edu.cn (X.-N.G.); 2020805112@stu.njau.edu.cn (X.-L.Z.); 2020105038@stu.njau.edu.cn (P.-Y.C.); zhangyanli@njau.edu.cn (Y.-L.Z.); caeet@njau.edu.cn (F.W.); 2 Animal Husbandry and Veterinary Station of Lianshui District, Huaian 223400, China; 2019105034@njau.edu.cn; 3 Perstorp Holding AB, 20125 Malmö, Sweden; ryen.wang@agrifirm.com

**Keywords:** Hu sheep, immune and antioxidant, negative energy balance, perinatal period, tributyrin

## Abstract

This study investigated the effects of tributyrin supplementation on the immune and antioxidant status of lambs born to perinatal Hu ewes with negative energy balance (NEB) at 75%. Twenty healthy Hu ewes (average body weight 45.00 ± 5.00 kg) with similar body condition score, pregnant for 100 days with twin lambs, were randomly allocated into two groups: NEB group and 0.5% tributyrin supplementation group (TB). Lambs were artificially fed with milk from their respective maternal groups for 42 days and were classified as L-NEB (*n* = 20) and L-TB (*n* = 20). The experimental period lasted from 40 days before delivery to 42 days after delivery. At 42 days of age, five male lambs from each group were randomly selected and euthanized for analysis. Tributyrin supplementation significantly increased fat-corrected milk yield, milk fat content, and colostrum fat yield in perinatal ewes (*p* < 0.01). Lambs in the L-TB group exhibited improved growth performance compared with L-NEB lambs (*p* < 0.05). Serum triglyceride concentration at 7 days of age was 68.4% higher, and serum triglyceride and glucose concentrations at 42 days of age were 42.9% and 13.9% higher, respectively, in the L-TB group than in the L-NEB group (*p* < 0.05). In addition, the L-TB group showed a 39.2% lower serum IL-1β concentration at 7 days and significantly downregulated hepatic IL-8 mRNA expression at 42 days, together with enhanced antioxidant capacity (*p* < 0.05). These findings suggest that dietary tributyrin supplementation at 0.5% in perinatal undernourished ewes, applied under a controlled NEB feeding regimen, may improve milk composition and, consequently, enhance growth performance, immune status, and antioxidant capacity in lambs.

## Simple Summary

This study evaluated the effects of dietary tributyrin supplementation in perinatal ewes experiencing energy deficiency and its association with offspring health and growth. The results indicated that tributyrin supplementation was associated with improved milk nutritional characteristics and increased growth performance in lambs, along with more favorable energy-related blood metabolic profiles. In addition, lambs from supplemented ewes showed lower levels of inflammatory indicators and higher antioxidant capacity. Within the limitations of the present experimental design, these findings suggest that tributyrin supplementation may contribute to supporting metabolic adaptation, immune function, and growth in lambs under NEB conditions.

## 1. Introduction

The perinatal period poses significant nutritional challenges for ewes, as they should meet not only their own physiological demands but also the substantial requirements for fetal growth and subsequent lactation [1]. The nutritional needs of the fetus peak approximately three weeks before parturition [2]. However, during this period, dry matter intake of ewes significantly decreases by 10–30% due to the physical constraints imposed by the developing lambs [3], resulting in nutrient deficiency in ewes. Negative energy balance (NEB) prior to parturition is a common and serious nutritional stress in sheep [4,5]. Ensuring adequate energy supply and maintaining healthy liver function, particularly gluconeogenic capacity, are considered key to mitigating the adverse effects of NEB and alleviating excessive lipid mobilization [6].

Butyric acid, a short-chain fatty acid constituting 5–20% of ruminal volatile fatty acids in adult ruminants, plays crucial roles in rumen epithelial cell proliferation, intestinal development, and nutrient absorption [7]. When butyric acid is absorbed by rumen epithelial cells, about 90% of butyric acid is oxidized into ketone bodies for energy supply, which is used to promote the proliferation and differentiation of rumen cells. When butyric acid enters the intestine, it can not only promote the growth of small intestinal epithelial cells but also accelerate the development of intestinal villi and improve the absorption of nutrients. However, its practical application is limited by its volatility, odor, and short half-life [8,9]. Tributyrin, a triglyceride of butyric acid, serves as an efficient precursor. It possesses a longer half-life, is safe, convenient to handle, and odorless, making it a more suitable feed additive [10].

Although tributyrin has been studied and applied in dairy cows [5], poultry [11], and pigs [12], its use in sheep remains limited. Tributyrin could relieve heat stress and increase the production performance of dairy cows [13]. Dietary supplementation with tributyrin could increase egg albumen quality and improve reproductive tract function [6]. Tributyrin can improve the performance of weaning piglets by modulating and stabilizing the intestinal microbiota [14]. Newborn lambs depend entirely on maternal resources, such as glucose and minerals supplied via colostrum and milk, for rapid early growth [15,16,17]. In prolific breeds like Hu sheep, which often bear twins, ensuring adequate nutrient transfer is particularly critical. Nutritional status of the ewe directly influences production and quality of colostrum and milk [18,19].

Therefore, this study aimed to determine whether dietary supplementing undernourished ewes with tributyrin could improve the growth performance, immune status, and antioxidant capacity of newborn lambs. While previous research has documented various benefits of tributyrin, systematic evidence regarding its comprehensive effects on lamb growth and immunity and antioxidant status when supplied to ewes during the perinatal period and subsequently transferred via milk is still lacking. We hypothesized that tributyrin supplementation would lead to positive outcomes in these aspects in their offspring. This study provides preliminary evidence that maternal tributyrin supplementation during the perinatal period is potentially associated with altered ewe milk quality as well as improved early-life growth, immune competence, and antioxidant capacity in lambs under NEB conditions.

## 2. Materials and Methods

### 2.1. Animals

Twenty Hu sheep (initial body weight 45.00 ± 5.00 kg, age = 2.50 ± 0.50 years) with good health reproductive performance, synchronized for estrus and artificially inseminated, were selected. The average gestation length was 100 ± 1 days, and all ewes carried twin fetuses. Ewes were randomly allocated to two groups (*n* = 10 per group) using a simple randomization procedure based on a random number generator, ensuring similar mean body weight and body condition between groups: control group (Negative Energy Balance Group: NEB group, 0.5% Tributyrin Supplementation: TB Group). Ewes were housed and fed individually. The tributyrin sample was provided by Perstorp Animal Nutrition (Prophorce SR130, Waspik, The Netherlands, hydrolyzed butyric acid ≥51.4%). Lambs born to ewes from NEB and TB groups were designated as L-NEB and L-TB, respectively.

### 2.2. Experimental Design and Diets

The trial consisted of a preliminary 10 days dietary adaptation period, followed by 82 days measurement period (from 40 days before birth to 42 days after birth). NEB and TB group ewes were fed a basal pelleted diet, provided twice daily at 08:00 and 14:00. The diet ingredients and nutritional indexes are shown in Table 1. They received an identical basal pelleted diet and were maintained under the same negative energy balance (NEB) feeding protocol throughout late pregnancy and lactation. Feed allowance was restricted to achieve an energy intake at approximately 75% of the National Research Council’s recommendations [20], while the dietary protein level was formulated to meet maintenance requirements. Thus, the NEB model was established primarily through energy restriction rather than protein deficiency. The diet for the TB group was prepared by supplementing the NEB basal diet with 0.5% tributyrin (dry matter basis) and proportionally reducing the basal ingredients to ensure equivalent nutrient composition between the two dietary treatments. The inclusion level of tributyrin (0.5% of diet DM) was selected with reference to previous studies in non-ruminant livestock showing effective and well-tolerated supplementation, together with the established biological roles of butyrate in ruminant metabolism [7,21]. Drinking water was provided ad libitum. Ewe milk was collected at 06:00, 10:00, 14:00, 18:00, and 22:00 each day. After collection, the milk was immediately refrigerated at 4 °C and used for lamb feeding within 24 h to prevent microbial spoilage. Lambs were fed with milk from their respective maternal groups, and the daily milk allowance was adjusted according to lamb body weight and age, following standard artificial rearing protocols, to ensure adequate and comparable nutrient intake among individuals. At the age of 42 days, five male lambs were randomly selected from each group and slaughtered for further analysis. The number of lambs selected for slaughter (*n* = 5 per group) was chosen to balance experimental feasibility and animal welfare while allowing tissue-level biochemical and gene expression analyses.

### 2.3. Analyses of Nutrient Apparent Digestibility

Between 34 and 41 days of age, five lambs from each group were individually housed in metabolic cages with free access to fresh water. Animals were allowed a 3-day adaptation period to the metabolic cages before the formal collection period. The metabolic cages were designed to allow complete separation of feces and urine, thereby minimizing the risk of cross-contamination. During the 5-day collection period, feces and urine were collected at the same time each morning. A urine collection device (latex funnel connected to a tube) was fitted to each lamb according to sex to ensure separate urine collection, and the urine was directed into plastic containers containing an appropriate amount of 50% sulfuric acid. Urine volume and feces weight were measured daily at fixed times. Ten percent of the total daily urine and feces were sampled, and composite samples over the 5-day period were prepared for each lamb. Sub-samples of urine and feces were stored at −20 °C. Before chemical analysis, feed and fecal samples were first dried in a forced-air oven at 65 °C for 48 h to a constant weight and then ground to pass through a 1-mm sieve. Ash content was determined by incinerating the dried samples in a muffle furnace (SX2-4-10, Shanghai Yiheng Scientific Instrument Co., Ltd., Shanghai, China) at 550 °C for 6 h. The nitrogen content was measured using the micro-Kjeldahl method with an automatic Kjeldahl analyzer (Kjeltec™ 8400, FOSS Analytical, Hillerød, Denmark). Crude protein concentration was calculated as nitrogen × 6.25. The gross energy of feed and fecal samples was determined by oxygen bomb calorimetry using a micro-automatic calorimeter (Parr 6400, Parr Instrument Company, Moline, IL, USA).

### 2.4. Sample Collection

Colostrum samples were collected from ewes within 24 h after parturition (first-day colostrum) for the analysis of colostrum composition and yield, and they were immediately stored at −80 °C in an ultra-low temperature freezer (DW-86L388J, Haier Bio-Medical, Qingdao, China) for further analysis. Blood samples were collected at 07:00 on days 7 and 42 after an overnight fast (approximately 12 h), and all animals were sampled at the same time point before the morning feeding. Blood was collected into anticoagulant Vacutainer, then cooled and centrifuged at 3000× *g* for 10 min. The serum was transferred to a 2 mL centrifuge tube and stored in a −80 °C refrigerator for further analysis. At the age of 42 days, five lambs were randomly selected from each group for slaughter after a 12-h fast (water ad libitum). Pre-slaughter live weight, carcass weight, and visceral organs’ (heart, liver, spleen, lung, kidney) weight and body fat were recorded. Immediately after slaughter, 20 g liver samples were collected and stored in a −80 °C refrigerator for the detection of immunity and anti-oxidation-related indexes.

### 2.5. Analyses of Serum Metabolites, Antioxidant and Immune Parameters

Glucose, triglyceride, total cholesterol, high density lipoprotein cholesterol, low density lipoprotein cholesterol, albumin, globulin, aspartate aminotransferase, alanine aminotransferase, serum alkaline phosphatase, and lactate dehydrogenase were measured using an automatic biochemical analyzer (AU 5800, Olympus, Tokyo, Japan). Commercial ELISA kits (Nanjing Jiancheng Bioengineering Institute, Nanjing, China) were used to determine the activities of superoxide dismutase (SOD, kit no. A001), catalase (CAT, kit no. A007), and glutathione peroxidase (GSH-Px, kit no. A005) in the samples, as well as the levels of tumor necrosis factor-α (TNF-α, kit no. H052), interleukin-4 (IL-4, kit no. H005), and interleukin-6 (IL-6, kit no. H007). Briefly, serum samples were thawed on ice and centrifuged at 1500 rpm for 5 min at 4 °C to remove any particulate matter before analysis. All procedures were performed strictly according to the manufacturers’ instructions. First, standard wells, sample wells, and blank wells were set up on a detection plate. A total of 50 μL of standard solutions with different concentrations were added to the standard wells. Then, add 10 µL testing samples and 40 µL sample diluent were added the sample wells. The blank wells received no sample or reagents. Secondly, 100 µL of the HRP-labeled detection antibody was added to each well and incubated for 60 min at 37 °C. Then, the wells were washed five times with washing solution, and 50 µL each of substrate A and B were added to every well. After, they were gently mixed and incubated for 15 min at 37 °C. Finally, the stop solution was added, and the OD nm values were read using a microtiter plate reader within 15 min.

### 2.6. Quantitative Real-Time Fluorescent Polymerase Chain Reaction

The total RNA of liver samples was extracted by Trizol reagent (Invitrogen, Carlsbad, CA, USA). The concentration and quality of the total RNA were determined with a Nanodrop 2000 spectrophotometry tool (Thermo Scientific, Waltham, MA, USA). When the absorption rate of all samples was between 1.8 and 2.0 at the OD 260/280, the total RNA samples were allowed to do further analysis. Total RNA was reverse transcribed into cDNA using the Primer Script RT reagent kit with gDNA eraser (TaKaRa Biotechnology, Dalian, China). The StepOne real-time PCR instrument (ABI QuantStudio5, Thermo Fisher Scientific, Waltham, MA, USA) was used to perform qPCR for all of genes with a commercial protocol of AceQ qPCR SYBR Green Master Mix (Vazyme, Nanjing, China). The National Center for Biotechnology (NCBI) was used to design primers online, and these primers were synthesized by Tsingke Biological Technology (Nanjing, China). The melting temperature, efficiency, and specificity of the primers were verified by PCR. The qPCR program consisted of an initial denaturation at 95 °C for 5 min, followed by 40 cycles of denaturation at 95 °C for 10 s, annealing at 60 °C for 30 s, and extension at 72 °C for 30 s, with a final extension at 72 °C for 7 min. The relative mRNA expression levels were calculated using the 2^−ΔΔCt^ method, in which gene expression was normalized to the reference gene and expressed relative to the control group [22], and the housekeeping gene beta actin (ACTB) was considered as endogenous control. The stability of ACTB was confirmed using GeNorm analysis (M value < 0.50). All of the gene tests were performed independently at least three times. The quantitative primers of different genes which were designed for programming qPCR are presented in Table 2.

### 2.7. Statistical Analysis

All data were tested for normal distribution using the Shapiro–Wilk test. Non-normal distribution data were log-transformed to achieve normality. All experimental data were analyzed using a one-way ANOVA in SPSS software (version24.0, SPSS Inc., Chicago, IL, USA). The results are presented as means and standard error of means (SEM), and all experiments were performed in triplicate. Statistical differences between treatment groups were further examined using Tukey’s test. Differences were considered statistically significant at *p* < 0.05, while 0.05 ≤ *p* < 0.10 was considered to indicate a tendency. In addition, the data shown in the graph were generated using GraphPad Prism version 8.0 (GraphPad Software, San Diego, CA, USA).

## 3. Results

### 3.1. Ewes and Lamb Production Performance

The growth performance and first-day colostrum quality of ewes are listed in Table 3. The body weight at weaning and average daily feed intake of TB ewes increased by 6.3% and 13.8%, respectively, compared with those of the NEB group (*p* = 0.037 and *p* = 0.026), while no difference was observed in initial body weight between groups (*p* = 0.546). Regarding colostrum composition, colostrum fat content in the TB group was higher by 318.1% compared with the NEB group (*p* < 0.001), whereas colostrum protein content was not affected (*p* = 0.546). Colostrum lactose content showed an increasing trend of 34.8% in the TB group (*p* = 0.077). In terms of colostrum yield, fat-corrected colostrum yield and colostrum fat yield in the TB group were higher by 142.9% and 307.7%, respectively, than those in the NEB group (both *p* < 0.001). Energy-corrected colostrum yield showed an increasing tendency of 90.7% (*p* = 0.056), while colostrum protein yield and lactose yield did not differ between groups (*p* = 0.546 and *p* = 0.077, respectively). As shown in Table 4, lambs from ewes fed tributyrin had 27.4% greater weaning weight, 29.4% higher average daily gain, and 7.4% greater average daily milk intake (ADMI) than L-NEB lambs (*p* = 0.030, *p* = 0.037, and *p* = 0.018, respectively). The live weight before slaughter and carcass weight were increased by 41.1% and 44.3%, respectively, in the L-TB group compared with the L-NEB group (*p* = 0.021 and *p* = 0.023). The dressing percentage of the L-TB group was 4.2% higher than that of the L-NEB group, showing an increasing tendency (*p* = 0.088).

### 3.2. Apparent Digestibility

Dietary apparent digestibility data are summarized in Table 5. Compared with the L-NEB group, the digestibility of crude protein and crude ash in the TB group increased by 7.6% (*p* = 0.005) and 14.5% (*p* = 0.003), respectively. The gross energy digestibility in the TB group was 12.8% higher than that in the L-NEB group (*p* = 0.032).

### 3.3. Serum Biochemical Parameters

The serum biochemical parameters of lambs at 7 and 42 days of age are shown in Table 6. At 7 days of age, serum glucose concentration did not differ between groups (*p* = 0.537). Compared with the L-NEB group, lambs in the L-TB group showed higher serum triglyceride, total cholesterol, and high-density lipoprotein cholesterol concentrations, which increased by 68.4% (*p* = 0.006), 44.1% (*p* = 0.002), and 20.8% (*p* = 0.002), respectively. Serum low-density lipoprotein cholesterol and globulin concentrations were not affected (*p* = 0.859 and *p* = 0.523), while albumin concentration showed a decreasing trend of 5.6% (*p* = 0.087). The activities of aspartate aminotransferase, alanine aminotransferase, alkaline phosphatase, and lactate dehydrogenase at 7 days of age were lower in the L-TB group, decreasing by 19.6% (*p* = 0.048), 44.0% (*p* = 0.017), 29.7% (*p* = 0.013), and 19.8% (*p* = 0.003), respectively. At 42 days of age, serum glucose and albumin concentrations in the L-TB group were higher than those in the L-NEB group, increasing by 13.9% (*p* = 0.021) and 14.2% (*p* = 0.011), respectively. Serum triglyceride and total cholesterol concentrations also increased by 42.9% (*p* = 0.013) and 26.7% (*p* = 0.011). High-density lipoprotein cholesterol content showed an increasing trend of 15.9% (*p* = 0.056), whereas low-density lipoprotein cholesterol and globulin concentrations were not significantly affected (*p* = 0.659 and *p* = 0.687). Lactate dehydrogenase activity remained lower at 42 days of age, showing a reduction of 35.7% in the L-TB group (*p* < 0.001).

### 3.4. Immune Indexes

**Figure 1:**
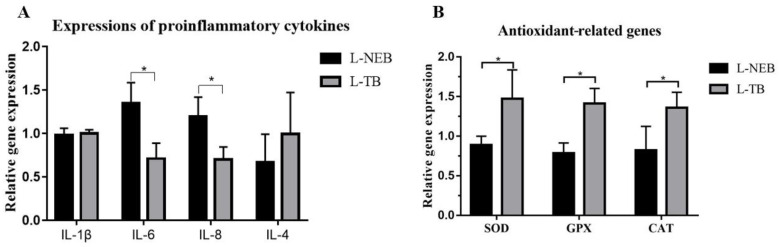
Effects of maternal tributyrin supplementation on the expression of liver immunity-related genes IL-1β, IL-6, IL-8, IL-4 and (**A**) antioxidant-related genes SOD, GSH-Px and CAT (**B**). L-NEB = Lambs in Negative Energy Balance Group; L-TB = Lambs in Tributyrin Group. Asterisks (*) above the error bars indicate significant differences between the two groups (*p* < 0.05), while the absence of an asterisk indicates no significant difference (*p* > 0.05). Normalized to ACTB, 2^−ΔΔCt^, fold-change relative to L-NEB (control).

The serum and liver immune indices of lambs are presented in Table 7. At 7 days of age, compared with the L-NEB group, serum interleukin-10 (IL-10) concentration increased by 51.0% (*p* < 0.001), whereas serum interleukin-1β (IL-1β) concentration was reduced by 39.2% (*p* < 0.001) in the L-TB group. At 42 days of age, serum IL-10 concentration was 19.3% higher (*p* < 0.001), while serum IL-1β concentration was 21.1% lower (*p* = 0.002) in the L-TB group compared with the L-NEB group. At 42 days of age, liver IL-4 content was 25.3% higher (*p* = 0.049), whereas liver IL-6 content was 11.6% lower (*p* = 0.028) in the L-TB group compared with the L-NEB group. As shown in Figure 1A, compared with the L-NEB group, maternal tributyrin supplementation significantly down-regulated the hepatic mRNA expression of IL-6 and IL-8 (*p* < 0.05).

### 3.5. Antioxidant Indices

The serum and liver antioxidant indices of lambs at 7 days and 42 days of age are listed in Table 8. Compared with the L-NEB group, the contents of serum superoxide dismutase (SOD), glutathione peroxidase (GSH-Px), and catalase (CAT) in the L-TB group were increased by 117.9%, 14.5%, and 72.8%, respectively, at 7 days (*p* < 0.001 for all), and by 49.5%, 33.8%, and 32.3%, respectively, at 42 days (*p* < 0.01 for all). At 42 days of age, the contents of liver SOD and CAT in the L-TB group were 78.6% (*p* < 0.001) and 42.0% (*p* = 0.015) higher, respectively, than those in the L-NEB group, whereas liver GSH-Px content showed an increasing tendency of 28.9% (*p* = 0.063). As shown in Figure 1B, the L-TB group exhibited higher hepatic mRNA expression of SOD, GSH-Px and CAT than the L-NEB group (*p* < 0.05).

## 4. Discussion

Poor nutritional status in perinatal and lactating ewes adversely affects fetal development and offspring growth [23]. Energy deficiency and the resulting body fat mobilization cause hypoglycemia and hyperketonemia, which are the main characteristics of NEB [24]. Perinatal ewes with low nutritional states are likely to negatively impact the growth and development of their lambs. In recent years, an intervention strategy termed “developmental programming” has been creatively applied to regulate the nutritional relationship between mothers and offspring primarily by modulating maternal nutrition to influence offspring development [25,26,27,28].

In the present study, as expected, ewes supplemented with tributyrin exhibited improved milk quality compared with NEB ewes, and the growth performance (weaning weight, ADG, LWBS, and carcass weight) of L-TB lambs was significantly higher than that of L-NEB lambs. Specifically, tributyrin supplementation increased milk fat content, fat yield, and energy-corrected milk production, indicating an enhanced energy supply to lambs during early life. This improvement in milk chemical composition may partly explain the superior growth performance observed in L-TB lambs. Consistent with a previous study, tributyrin supplementation increased the growth performance of dairy calves after weaning [29], but there was no effect on the growth (BW, ADG, and structural growth) during the pre-weaning period, which differs from our findings. These findings indicate that maternal tributyrin supplementation exerts measurable effects on offspring growth primarily through changes in milk quality.

In addition, another study showed that tributyrin supplementation negatively affected feed intake and ADG in pre-weaning calves [30]. These discrepancies may be partly explained by differences in supplementation strategy and duration. In contrast to the direct feeding of tributyrin to calves in previous studies, tributyrin in the present study was provided to ewes during the perinatal period, thereby influencing lamb development through maternal metabolism and milk-mediated transfer. Tributyrin is rapidly hydrolyzed to butyrate in the rumen and intestine of ewes, and the resulting butyrate is absorbed into the circulation and partially utilized by peripheral tissues, including the mammary gland [21]. Therefore, maternal tributyrin supplementation may influence lamb growth primarily through maternal metabolism and milk-mediated nutrient transfer rather than through direct supplementation to offspring. The significant increase of triglyceride content in serum may result from the glycerol obtained after the decomposition of tributyrin [31]. Previous studies have shown that adding glycerol to daily rations can significantly increase the content of VFA after ruminants eat, and the increase of VFA content can further increase the content of glucose in serum, which is consistent with our experimental results [6,32]. These differences may also reflect the indirect nature of maternal supplementation, whereby tributyrin first modulates maternal metabolism and milk composition before affecting the offspring, in contrast to the more direct exposure associated with supplementation in young animals.

To better understand the immunological basis underlying these phenotypic changes, we focused on cytokines, key mediators produced by immune and nonimmune cells that regulate inflammatory responses, including pro-inflammatory (IL-1β, IL-6, IL-8) and anti-inflammatory cytokines (IL-4, IL-10) [33]. We found that tributyrin supplementation to ewes during the perinatal period significantly reduced the amount of pro-inflammatory cytokines in lamb serum and mRNA expression in lamb liver at different periods, contrary to anti-inflammatory cytokines.

In this study, we found that the content of IL-1β in the serum of 7 days and 42 days lambs and the mRNA expression of IL-6 and IL-8 in the livers of 42 days lambs were significantly decreased after the supplementation of tributyrin. IL-6 can mediate the destruction of various immune barriers by activating matrix metalloproteinase 9, which leads to the increase of barrier permeability, facilitates the invasion of bacteria and inflammatory cells, and promotes the development of various inflammation [34]. The main functions of IL-6, IL-1β, and IL-8 includes promoting the development of inflammation, and IL-1β and IL-6 are endogenous sources of heat, which can cause fever [35]. Guo et al. found that tributyrin could significantly reduce the gene expression of IL-6 and IL-1β in the peripheral blood lymphocytes of dairy cows and inferred that tributyrin could reduce the inflammatory response of peripheral blood lymphocytes of dairy cows [13]. These findings coincide with our findings.

It has been reported that a persistent association between umbilical cord inflammation and elevated circulating inflammatory proteins at post-natal day 7 may contribute to sustained systemic inflammation, which has been linked to conditions such as septicemia, bronchopulmonary dysplasia, and necrotizing enterocolitis [36]. We found that the IL-10 content in the serum of 7 days lambs and the mRNA expression of IL-4 in 42 days lambs were significantly increased after the supplementation of tributyrin. The upregulation of these anti-inflammatory cytokines indicates that lambs in the L-TB group exhibited a greater capacity to suppress excessive inflammatory responses associated with fetal inflammatory response syndrome [37,38,39]. Taken together, these findings suggest that maternal tributyrin supplementation may contribute to a more balanced inflammatory environment during early life in lambs.

From a mechanistic perspective, tributyrin is rapidly hydrolyzed to butyrate in the rumen and intestine of ewes [40]. The resulting butyrate serves as both an energy substrate and a signaling molecule in mammary epithelial cells [41,42]. Additionally, glycerol produced from tributyrin hydrolysis can contribute to glucose availability for lactose synthesis [43,44]. These metabolites may be transferred into milk via maternal circulation, thereby enhancing milk fat content and energy-corrected milk yield [45]. Consequently, maternal tributyrin supplementation likely improves milk quality by modulating both mammary gland metabolism and systemic energy supply. The differences between our results in periparturient ewes and previous studies in dairy calves or piglets may be due to species-specific characteristics, developmental stage, and the maternal rather than direct supplementation approach.

The higher activities of ALT, ALP, and LDH in L-NEB lambs indicate liver stress and cell injury caused by energy deficiency during early life [46]. In contrast, the lower levels of these enzymes in L-TB lambs suggest that maternal tributyrin supplementation helped protect liver function. This effect may be associated with a more stable metabolic state and reduced inflammatory burden, which together could help limit liver damage under a negative energy balance.

In our study, we found that the contents of SOD, GSH-Px, and CAT in the serum of 7 days and 42 days lambs significantly increased after tributyrin supplementation. In addition, the hepatic mRNA expression of SOD, GSH-Px, and CAT in 42 days lambs was significantly upregulated. Although hepatic GSH-Px activity in the L-TB group showed only an increasing trend, its mRNA expression was significantly increased. This indicates that tributyrin enhanced the production of GSH-Px at the gene level, which likely contributed to the improved antioxidant capacity observed in the liver of L-TB lambs. CAT, SOD, and GSH-Px are the main enzymes that play an antioxidant role in the body, and the increase of their activity can effectively prevent the injury caused by oxidative stress [47]. Reactive oxygen superoxide anion changes into oxygen and hydrogen peroxide under the action of SOD, hydrogen peroxide decomposes into water under the action of CAT, and GSH-Px can scavenge peroxides [48,49,50]. Through their coordinated actions, these enzymes effectively reduce cellular damage caused by reactive oxygen species [51]. Consistent with our results, previous studies have shown that tributyrin supplementation improved antioxidant capacity in broiler breeders’ ovary and magnum; this may also be the possible reason for the improving effects of tributyrin on albumen quality [6]. Similarly, another study showed that sodium butyrate supplementation could improve antioxidant function (increase glutathione peroxidase activity) in pre-weaning cows [52].

This study has several limitations. The number of lambs selected for slaughter was relatively small (*n* = 5 per group). This sample size was determined by practical feasibility and animal welfare considerations and is comparable to that used in previous ruminant studies involving destructive tissue sampling; nevertheless, caution is warranted when interpreting tissue-level biochemical and gene expression data. In addition, only a single level of tributyrin supplementation (0.5%) was evaluated, which prevented the assessment of potential dose–response relationships. Although this inclusion level was chosen based on prior evidence of efficacy and tolerability, the observed effects should be interpreted as specific to this dosage. Maternal gut microbiota and related metabolic profiles were not examined in the present study, despite their recognized roles in ruminal fermentation and nutrient utilization. Moreover, tributyrin- or butyrate-derived metabolites were not directly quantified in ewe milk or lamb tissues, and therefore maternal–offspring transfer can only be inferred. Future studies using multiple supplementation levels and directly measuring milk and tissue metabolites may help provide a more complete understanding of the present observations.

## 5. Conclusions

Maternal tributyrin supplementation at 0.5% during the perinatal period under negative energy balance (NEB) conditions was associated with higher milk fat yield and energy-corrected milk in ewes, along with improved growth performance, metabolic status, immune balance, and antioxidant capacity in their lambs. Lambs born to tributyrin-supplemented ewes showed higher concentrations of energy-related metabolites, lower inflammatory markers, and more favorable indicators of liver function. Overall, these results suggest that maternal tributyrin supplementation may support milk composition and early-life metabolic and immune adaptation in lambs under NEB conditions.

## Figures and Tables

**Table 1:** Experimental diet composition and nutrient profile.

Items	Late Pregnancy Feed ^1^		Lactation Feed ^1^	
	NEB	TB	NEB	TB
Ingredients (g/kg DM)				
Corn	150.00	149.00	180.00	179.10
Premix ^2^	50.00	49.80	50.00	49.80
Rice husk	250.00	249.00	190.00	189.10
Soybean husk	230.00	229.00	220.00	218.90
Corn germ meal	160.00	159.00	160.00	159.20
Soybean meal	120.00	119.40	160.00	159.20
Rice bran	40.00	39.80	40.00	39.70
Tributyrin	00.00	5.00	00.00	5.00
Total	1000.00	1000.00	1000.00	1000.00
Chemical composition				
Metabolic energy, MJ/kg	2.18	2.18	2.28	2.28
Crude protein, g/kg DM	141.50	141.50	176.30	176.30
Ether extract, g/kg DM	29.10	29.10	30.50	30.50
Ash, g/kg DM	81.70	81.70	62.60	62.60
Ca, g/kg DM	10.20	10.20	9.90	9.90
P, g/kg DM	5.10	5.10	5.40	5.40

^1^ NEB = Negative energy balance group; TB = Tributyrin group. ^2^ Premix (per kg) contains: vitamin A, 120,000 IU; vitamin D3, 35,000 IU; vitamin E, 4000 mg; nicotinamide, 1200 mg; Cu, 160 mg; Na, 25 g; Fe, 600 mg; Co, 10 mg; Mn, 750 mg; Se, 6 mg; Zn, 800 mg; I, 8 mg; H_2_O, 100 g.

**Table 2:** Primer sequences and amplicon information.

Gene	Primers (5′–3′)	Product Length (bp)	Accession
SOD	F: TCAATAAGGAGCAGGGACGC	209	NM_001280703.1
	R: GCTGCAAGCTGTGTATCGTG		
GSH-Px	F: ACATTGAAACCCTGCTGTCC	178	JF728302.1
	R: TCATGAGGAGCTGTGGTCTG		
CAT	F: TGGGACCCAACTATCTCCAG	216	XM_004016396.3
	R: AAGTGGGTCCTGTGTTCCAG		
IL-1β	F: ACAGATGAAGAGCTGCACCC	161	XM_013967700.2
	R: AGACATGTTCGTAGGCACGG		
IL-6	F: CACCACCCCAAGCAGACTAC	218	NM_001285640.1
	R: AGCAAATCGCCTGATTGAACC		
IL-8	F: TGTGTGAAGCTGCAGTTCTGT	186	XM_005681749.3
	R: TGGGGTCTAAGCACACCTCT		
IL-2	F: CAAGCTCTACGGGGAACACA	147	NM_001287567.1
	R: CTGTAGCGTTAACCTTGGGC		
IL-4	F: CGCTGAACATCCTCACATCG	170	NM_001009313.3
	R: AAGTCCGCCCAGGAATTTGT		
ACTB	F: CGCAAGTACTCCGTGTGGAT	146	NM_001009784.3
	R: TAACGCAGCTAACAGTCCGC		

F = Forward primer; R = reverse primer; SOD = superoxide dismutase; GSH-Px = glutathione peroxidase; CAT = catalase; IL-1β = interleukin-1 beta; IL-2 = interleukin-2; IL-4 = interleukin-4; IL-6 = interleukin-6; IL-8 = interleukin-8; ACTB = beta-actin; bp = base pairs.

**Table 3:** Effects of tributyrin supplementation during the perinatal period on growth performance and first-day colostrum quality of ewes.

Items ^1^	Groups ^2^		SEM	*p*-Value
	**NEB**	**TB**		
Initial BW, kg	45.32	44.69	4.89	0.546
BW at lamb weaning, kg	36.62	38.92	4.23	0.037
ADFI, kg/d	1.38	1.57	0.32	0.026
Colostrum composition				
Fat, %	2.27	9.49	1.63	<0.001
Protein, %	7.17	7.95	0.60	0.546
Lactose, %	1.15	1.55	0.12	0.077
Colostrum composition yield				
Fat yield, kg/d	0.01	0.05	0.01	<0.001
Protein yield, kg/d	0.04	0.05	0.00	0.546
Lactose yield, kg/d	0.01	0.01	0.00	0.077
FCM, kg/d	0.42	1.02	0.14	<0.001
ECM, kg/d	0.54	1.03	0.13	0.056

Values represent measurements obtained from first-day colostrum. ^1^ FCM = fat-corrected milk; ECM = energy-corrected milk. ^2^ NEB = Negative Energy Balance Group; TB = 0.5% Tributyrin Supplementation Group. SEM = standard error of the mean; BW = body weight; ADFI = average daily feed intake.

**Table 4:** Effects of maternal tributyrin supplementation on offspring growth and slaughter performance.

Items ^1^	Groups ^2^		SEM	*p*-Value
	L-NEB	L-TB		
Growth performance				
BW, kg	3.22	3.63	0.29	0.412
Weaning weight, kg	10.85	13.82	0.73	0.030
ADG, kg	0.17	0.22	0.13	0.037
ADMI, g/d	214.22	230.13	9.20	0.018
Slaughter performance				
LWBS, kg	11.28	15.92	1.09	0.021
Carcass weight, kg	5.64	8.14	0.59	0.023
Dressing percentage, %	49.67	51.76	0.61	0.088
GR value, mm	6.00	8.42	1.11	0.301

^1^ BW = birth weight; ADG = average daily gain; ADMI= average daily milk intake; LWBS = live weight before slaughter; GR = the tissue depth between the 12th and 13th ribs 11 cm from the midline of the carcass’s spine; ^2^ L-NEB = lambs in Negative Energy Balance Group; L-TB = lambs in Tributyrin Group.

**Table 5:** Effects of maternal tributyrin supplementation on apparent digestibility of nutrients in lambs.

Items	Groups		*p*-Value
	L-NEB	L-TB	
Crude protein (%)	14.83 ± 0.41	15.96 ± 0.39	0.005
Crude ash (%)	19.25 ± 0.61	22.47 ± 0.49	0.003
Gross energy (%)	11.63 ± 0.76	13.12 ± 0.59	0.032

**Table 6:** The effects of maternal tributyrin supplementation on serum biochemistry in lambs.

Items ^1^	Groups ^2^		SEM	*p*-Value
	L-NEB	L-TB		
Day 7				
Glucose, mmol/L	6.16	6.56	0.28	0.537
TG, mmol/L	0.57	0.96	0.08	0.006
TC, mmol/L	1.61	2.32	0.14	0.002
HDL-C, mmol/L	1.01	1.22	0.05	0.002
LDL-C, mmol/L	0.56	0.57	0.03	0.859
Albumin, g/L	28.75	27.14	0.47	0.087
GLO, g/L	34.25	36.81	1.83	0.523
AST, U/L	72.43	60.85	2.99	0.048
ALT, U/L	9.17	5.14	0.90	0.017
ALP, U/L	1265.67	890.14	81.20	0.013
LDH, U/L	697.60	559.00	27.13	0.003
Day 42				
Glucose, mmol/L	5.06	5.77	0.17	0.021
TG, mmol/L	0.49	0.70	0.05	0.013
TC, mmol/L	1.91	2.42	0.38	0.011
HDL-C, mmol/L	1.13	1.31	0.05	0.056
LDL-C, mmol/L	0.61	0.65	0.04	0.659
Albumin, g/L	29.16	32.14	0.65	0.011
GLO, g/L	29.51	30.40	0.99	0.687
AST, U/L	62.17	72.67	4.30	0.239
ALT, U/L	6.00	7.67	0.53	0.123
ALP, U/L	658.67	705.00	37.98	0.567
LDH, U/L	674.17	433.50	39.00	<0.001

^1^ TG = triglyceride; TC = total cholesterol; HDL-C = high-density lipoprotein cholesterol; LDL-C = low-density lipoprotein cholesterol; GLO = globulin; AST = aspartate aminotransferase; ALT = alanine aminotransferase; ALP = Alkaline Phosphatase; LDH = lactate dehydrogenase. ^2^ L-NEB = Lambs in Negative Energy Balance Group; L-TB = Lambs in Tributyrin Group.

**Table 7:** The effects of maternal tributyrin supplementation on immune indexes in lambs.

Items ^1^	Groups ^2^		SEM	*p*-Value
	L-NEB	L-TB		
Serum-related				
Day 7				
IL-1β, ng/L	61.59	37.42	3.11	<0.001
IL-10, ng/L	50.45	76.17	3.24	<0.001
Day 42				
IL-1β, ng/L	45.74	36.01	1.73	0.002
IL-10, ng/L	65.61	78.24	1.86	<0.001
Liver-related				
Day 42				
IL-4, ng/L	20.74	25.99	1.38	0.049
IL-6 ng/L	48.37	42.74	1.30	0.028
TNF-α, ng/L	136.06	133.75	2.32	0.632

^1^ IL = interleukin; TNF = tumor necrosis factor. ^2^ L-NEB = Lambs in Negative Energy Balance Group; L-TB = Lambs in Tributyrin Group.

**Table 8:** Effects of maternal tributyrin supplementation on antioxidant indices in lambs.

Items ^1^	Groups ^2^		SEM	*p*-Value
	L-NEB	L-TB		
Serum				
Day 7				
SOD, U/mL	85.05	185.31	12.18	<0.001
GSH-Px, U/mL	357.68	409.71	7.34	<0.001
CAT, U/mL	44.39	76.71	3.91	<0.001
Day 42				
SOD, U/mL	103.29	154.37	6.54	<0.001
GSH-Px, U/mL	243.65	325.65	10.95	<0.001
CAT, U/mL	54.19	71.66	2.19	<0.001
Liver				
Day 42				
SOD, U/mg	22.31	39.84	2.93	<0.001
GSH-Px, U/mg	4.47	5.76	0.35	0.063
CAT, U/mg	6.02	8.55	0.53	0.015

^1^ SOD = superoxide dismutase; GSH-Px = glutathione peroxidase; CAT = catalase. ^2^ L-NEB = Lambs in Negative Energy Balance Group; L-TB = Lambs in Tributyrin Group.

## Data Availability

The original contributions presented in this study are included in the article. Further inquiries can be directed to the corresponding author.

## Abbreviations

The following abbreviations are used in this manuscript:

NEB	Negative energy balance
TB	Tributyrin supplementation group
TG	The contents of serum triglyceride
NRC	National Research Council
SOD	Superoxide dismutase
CAT	Catalase
GSH-Px	Glutathione peroxidase
TNF-α	Tumor necrosis factor-α
IL-4	Interleukin-4
IL-6	Interleukin-6
OD	Optical density
DNA	Deoxyribonucleic acid
RNA	Ribonucleic acid
mRNA	Messenger RNA
qPCR	Quantitative real-time polymerase chain reaction
PCR	Polymerase chain reaction
USA	United States of America
NCBI	National Center for Biotechnology
SEM	Standard error of means
LWBS	The live weight before slaughter
FCM	Fat-corrected milk
ECM	Energy-corrected milk
BW	Body weight
ADFI	Average daily feed intake
ADG	Average daily gain
ADMI	Average daily milk intake
GR	The tissue depth between the 12th and 13th ribs 11 cm from the dorsal midline
TC	Total cholesterol
HDL-C	High-density lipoprotein cholesterol
LDL-C	Low-density lipoprotein cholesterol
GLO	Globulin
AST	Aspartate aminotransferase
ALT	Alanine aminotransferase
ALP	Alkaline phosphatase
LDH	Lactate dehydrogenase
IL	Interleukin
TNF	Tumor necrosis factor
